# A self-evolution cyber attack scheme generation system for cybersecurity evaluation

**DOI:** 10.1038/s41598-026-37012-0

**Published:** 2026-01-28

**Authors:** Mingsheng Yang, Yan Jia, Yangyang Mei, Jie Yang, Weihong Han, Jiawei Zhang, Zhuocheng Yu

**Affiliations:** Peng Cheng Laboratory, Department of New Networks, Shenzhen, 518000 China

**Keywords:** Computer science, Information technology

## Abstract

With the increasing complexity of network attacks, defense systems face significant challenges in maintaining cybersecurity. To effectively evaluate and optimize defense strategies, this paper proposes a self-evolution attack scenario generation system tailored for assessment purposes. To address the scalability challenges in attack graph generation and improve the efficiency and relevance of security evaluations, the system incorporates a real-time generation method capable of dynamically adapting attack scenarios based on specific goals and constraints. Additionally, a methodology is developed to construct potential attack paths using attack graph techniques enhanced with self-evolving mechanisms. The feasibility and adaptability of the generated attack scenarios are validated through simulation experiments. This paper details the system’s design, highlighting its core technical innovations-including incremental graph updates, scalable goal-driven path generation, and quantitative path ranking–which address key limitations of traditional tools like MulVAL. The system’s effectiveness and superiority in scalability and usability are demonstrated through extensive simulations.

## Introduction

In the highly digitalized contemporary society, network systems not only constitute essential infrastructure for enterprises and government agencies, but also serve as a crucial target for attackers to inflict damage and extract information. With the rapid advancement of cyber attack techniques, attackers are constantly adopting novel approaches to circumvent traditional security defense systems, leading to frequent occurrences of advanced persistent threats (APTs), ransomware attacks, and supply chain attacks. Here, single-stage attacks have evolved into multi-stage chain attacks, posing significant challenges for defenders to anticipate and prevent^1^. To increase the resilience and adaptability of network defense systems, numerous researchers have begun to focus on how to proactively simulate potential attack paths, thus identifying and strengthening system vulnerabilities at an early stage.

Traditional network security methods, such as firewalls and intrusion detection systems (IDS), are known for matching known threat signatures. However, these methods often struggle to detect unknown attacks or variants of known attacks^2^. To effectively identify multi-step attack paths and their associated generation systems have been developed. These systems simulate the attacker’s perspective to generate possible attack paths, helping security personnel gain insight into vulnerable areas of the system in advance, thus optimizing and strengthening the network security defense system. Attack scenario generation systems for evaluation purposes are different from traditional attack detection systems. They generate and simulate a series of complex attack paths to test the defense effectiveness of a network system when facing multi-stage attacks. In recent years, attack graphs have been widely used in network security evaluation and attack path generation as a model of network vulnerabilities^3^. Attack graphs represent the nodes and edges of a network system in graphical form, where nodes represent key components of the system (such as hosts, services, permissions, etc.), and edges represent the actions that attackers can take at different stages (such as exploiting vulnerabilities, elevating privileges, and lateral movement, etc.). Quite a lot of research has been conducted, proposing various methods to generate attack graphs^4–6^, optimizing methods^7^, and application analysis methods^8–11^. Based on attack graphs, the risk level of each network node can be calculated^12^, the critical nodes of the network can be identified^13^, the payoff of offensive and defensive strategies can be quantified^14^, the security of the network system can be evaluated^15,16^, and it is a commonly used method for evaluating network security.

In a network security evaluation system, the target range of the network is simulated and vulnerability scanning tools are used to identify weaknesses within the network. Based on these scan results, an attack graph is generated. By analyzing the graph, the attack paths for each node can be traced and the risk and exploitability of the information can be quantitatively assessed. This allows for the calculation of the risk level of each node and the overall risk assessment of the system^17,18^. Currently, attack graph analysis relies heavily on manual evaluation, which is inefficient and prone to inaccuracies. As the scale and complexity of the system grow, particularly with large network clusters, the attack graph becomes exponentially more complex, making it difficult to generate, analyze, and interpret. Furthermore, attack graphs, while powerful for modeling multi-step attacks, suffer from well-known scalability issues: the number of potential paths grows exponentially with network size, and graphs often contain redundant or irrelevant paths^19^. This “path explosion” problem makes manual analysis infeasible and limits the applicability of traditional tools like MulVAL in large-scale or dynamic networks.^19^

In summary, this paper presents a self-evolution attack scheme generation system for the evaluation system. By parsing the system vulnerability information, the attack-reachable host nodes within the system and the attack transfer state information between the host nodes are identified. With the hosts as nodes, they are presented to the users through a visualization system. The users can select specific targets of the system (such as key hosts) to prioritize the generation of paths, thereby automatically generating attack plans. It can also provide recommended attack steps and quantitative evaluation values for different attack paths in the attack plan. At the same time, the system will dynamically update the attack paths based on real-time vulnerability data and user-defined constraints (such as path thresholds, host/node filters). This enables security assessment personnel to conduct attack test verification and system risk assessment with the assistance of this system. The contributions obtained through the method proposed in this study are as follows:

- To quantitatively assess the information such as the risk and exploitability of attack paths, we collected various types of information for each node and edge in the network and constructed a vulnerability information management system.
- We designed a vulnerability information parsing method and an attack scheme generation method to ensure that the system can rapidly generate and optimize attack paths and exhibit excellent performance in complex network environments.
- We constructed a small-scale simulation network containing multiple nodes in a network range to verify that the proposed attack generation scheme outperforms the existing benchmark methods.

The remainder of this paper is organized as follows. In Sect. 2, the related research works are compared and analyzed. In Sect. 3, we introduce the relevant content of the attack scheme generation system framework based on attack graphs. In Section 4, we introduce the implementation methods of key technologies. In Sect. 5, we verify the designed system through simulation experiments. In Sect. 6, we discuss the limitations of this study and future research directions. Finally, we conclude and discuss the future work directions in Sec. 7.

## Related works

The field of attack graph generation and analysis is rapidly evolving. While foundational systems like MulVAL^20^^21^ [20] provide a logic-based framework for analysis, recent research has focused on enhancing scalability, integrating artificial intelligence, and applying these techniques to new domains like the Internet of Things (IoT) and industrial control systems.

Beyond the traditional MulVAL-based logical frameworks, numerous recent studies have investigated the scalability challenges of AGs. Nadeem et al.^22^ proposed S-PDFA-based alert-driven AG generation, reducing redundant expansion by constraining transitions to observed alerts. Sun et al.^23^ integrated heuristics for risk assessment and selective expansion. Terranova et al.^24^ employed deep reinforcement learning for predicting probabilistic attack paths, offering generalization to unseen network states. Palma and Angelini^25^ further introduced an analysis-driven, scalable AG generation framework supporting dynamic updates, demonstrating that selective recomputation can considerably mitigate scalability bottlenecks. In addition, recent surveys^26–28^ provide comprehensive analyses of MulVAL extensions, attack scenario coverage, and AG scalability benchmarks, emphasizing the lack of systems that combine both practical usability and dynamic behavior while effectively handling large network graphs.

Early work by Swiler et al.^4^ established the graph-based analysis of network vulnerabilities. Subsequent efforts by Kaynar & Sivrikaya^5^ addressed the challenge of distributed attack graph generation, while Hankin et al.^6^ developed frameworks for automatic generation by integrating system topology with standard databases like CAPEC, CWE, and CVE. Recent studies have pushed the boundaries further, exploring dynamic analysis, reinforcement learning for path discovery, and graph aggregation techniques for a more realistic assessment of complex networks. The following Table 1 provides a comparative overview of these recent studies, highlighting their core contributions and limitations in the context of automated attack path generation for cybersecurity evaluation.

**Table 1:** Comparative overview of recent studies on attack graph generation and analysis.

Reference	Focus / Contribution	Methodology / Approach	Key Limitation / Challenge Addressed
Almazrouei et al.^3^	Comprehensive review of AG analysis for IoT vulnerability assessment.	Survey of existing methods, trends, and open challenges in IoT security.	Highlights the lack of standardized AG frameworks tailored for heterogeneous IoT environments.
Boudermine et al.^19^	Dynamic, logic-based AG for real-time risk assessment in complex systems.	Extends traditional AGs with dynamic logic to model evolving system states and attacker actions.	Complexity of model reasoning increases significantly with system scale, impacting performance.
Mai et al.^29^	Report Analysis Framework for automated attack path generation (RAF-AG).	Parses and correlates data from diverse security reports to generate actionable attack paths.	Relies on the quality and format of input reports; may struggle with incomplete data.
Lyu et al.^30^	Multi-stage attack correlation and scenario reconstruction (AGCM) based on graph aggregation.	Uses graph aggregation techniques to correlate alerts and reconstruct entire attack scenarios.	The method’s efficacy is tied to the quality and completeness of the underlying alert data.
Nguyen et al.^15^	PenGym: A realistic RL training environment for penetration testing agents.	Leverages AGs to create a gym environment for training RL agents in multi-step attack strategies.	Focus is on training AI agents rather than on efficient AG generation or visualization for human analysts.
Nadeem et al.^22^	Alert-driven AG generation using S-PDFA.	Automates AG generation from IDS alerts.	Limited to alert-driven scenarios; not goal-oriented.
Sun et al.^23^	Heuristic risk assessment based on AG.	Combines AG with heuristic search for risk evaluation.	Does not support real-time incremental updates.
Terranova et al.^24^	DRL for attack path prediction.	Uses deep reinforcement learning to predict likely paths.	Focus on prediction, not comprehensive AG generation.
Palma & Angelini et al.^25^	Scalable analysis-driven AG generation.	Steers AG construction based on analysis goals.	Requires predefined analysis queries.
Tayouri et al.^26^	Survey of MulVAL extensions.	Comprehensive review of AG scalability solutions.	Highlights lack of integrated self-evolution mechanisms.
Palma & Bonomi et al.^27^	Vulnerable network generator for AG evaluation.	Provides benchmarks for AG scalability testing.	Tool-focused, not a complete AG system.
Our Work	A self-evolution attack scheme generation system for evaluation.	Integrates real-time parsing, grouping strategies, and path thresholding for scalable and interactive AG generation and visualization.	Addresses path explosion and manual analysis inefficiency through automated, user-constrained generation and intuitive visualization.

As illustrated in the table, contemporary research tackles various aspects of attack graph technology, from theoretical models [19] and application surveys [3] to AI-integrated frameworks [16, 22] and correlation methods [23]. However, a significant gap remains in creating systems that are both highly scalable and practically usable for security evaluators. Many approaches still grapple with the inherent complexity of AGs or focus on niche applications like AI training.

Compared with these studies, our work differs in three key ways:

1. While alert-driven and DRL-based methods^22,24^ focus on detection or prediction, our system is explicitly designed for evaluation, enabling human analysts to generate custom attack schemes under user-defined constraints.
2. Unlike dynamic logic-driven frameworks^25^, which primarily optimize symbolic reasoning, our approach integrates a practical host grouping + path-thresholding strategy that guarantees linear-time performance for goal-driven path generation.
3. Recent MulVAL-extension surveys^26,27^ highlight the lack of systems providing real-time, interactive, evaluation-oriented AG visualization. Our system fills this gap by providing incremental updates, intuitive visualization, and traceable attack-step recommendations.

Our proposed system directly addresses these challenges. Unlike PenGym^15^ which focuses on an AI training environment, our system is designed for human-in-the-loop security evaluation. Compared to the dynamic logic approach of Boudermine et al.^19^, we prioritize scalability and clarity by employing host grouping and path thresholding to manage complexity, ensuring the system remains responsive and interpretable even as the network grows. By providing an interactive visualization interface and allowing users to define constraints (e.g., target hosts, path limits), we offer a practical tool that bridges the gap between fully automated analysis and manual expert assessment, enabling efficient and relevant cybersecurity evaluation.

**Figure 1:**
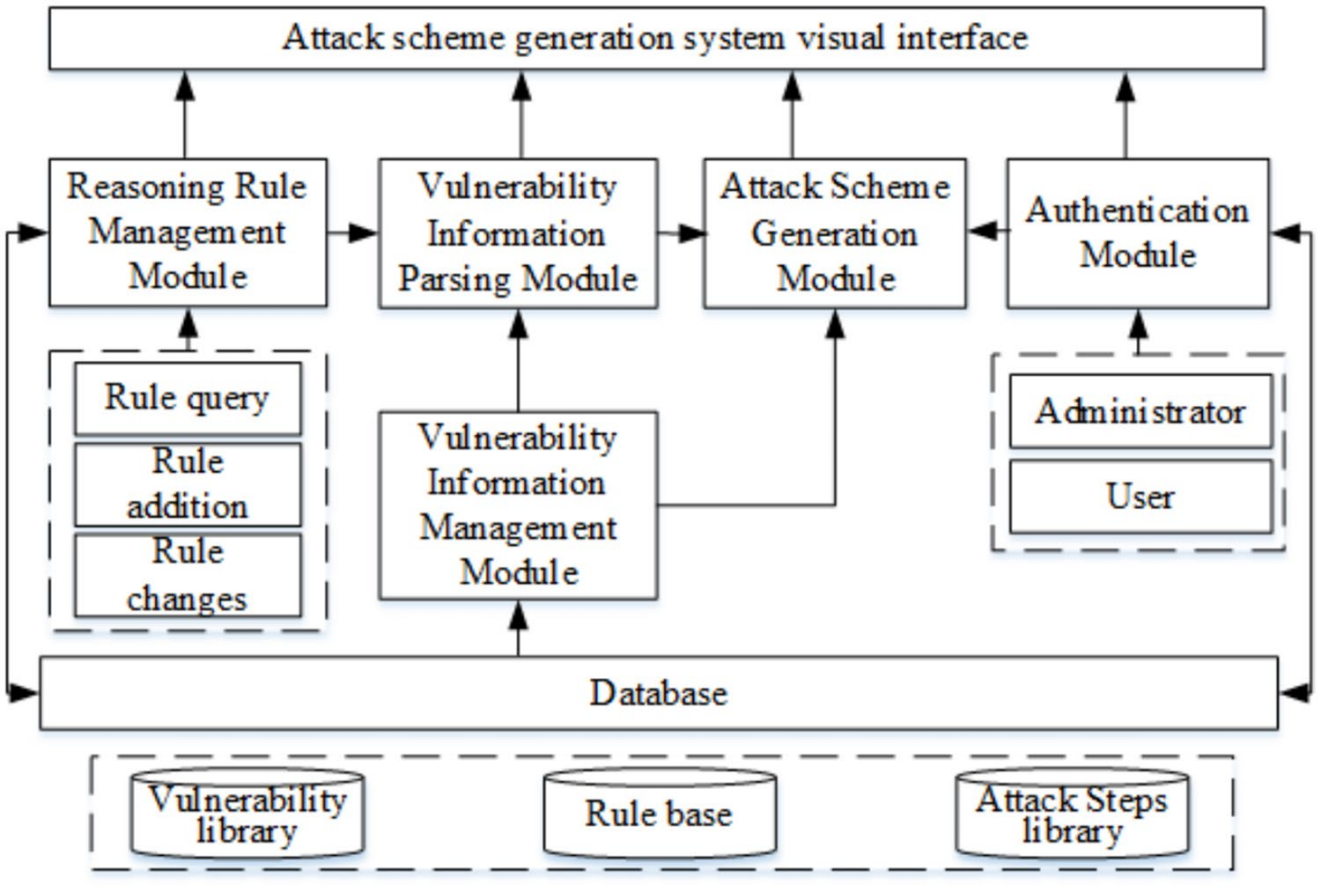
Architecture diagram of attack scheme generation system.

## Framework of the attack scheme generation based on the attack graph

The attack scheme generation system oriented to assessment mainly consists of the reasoning rule management module, vulnerability information management module, vulnerability information parsing module, attack scheme generation module, user interaction visualization module, and database. The system framework is depicted in Fig. 1. The design objective of the system is to automatically generate and optimize the attack paths and verify the feasibility and threat to the system of these paths in a simulation environment. The system enhances its usability and scalability through modular design, enabling different modules to develop, and improve independently.

### Reasoning rule management module

The reasoning rule management module controls the reasoning rules used to parse vulnerability information, including exploit rules, threat propagation rules, and multi-hop network access rules. It provides interfaces for querying, updating, and modifying these rules to meet various assessment needs. The system comes pre-configured with a default set of reasoning rules that cover common attack behaviors and vulnerability exploitation paths, which are automatically parsed from public databases such as CAPEC and CVE. These rules are stored in a structured format (Datalog) and can be directly invoked without manual intervention. To satisfy diverse attack scenarios and evaluation demands, users can also create and define customized rules to adapt to specific network environments or alterations in security policies. While expert input may enhance rule specificity, the system minimizes human effort through automated rule extraction and template-based rule generation. The flexible configuration and scalability of reasoning rules enhance the adaptability of the system in complex network environments.

### Vulnerability information management module

The vulnerability information management module handles the storage and management of vulnerability and attack procedure data within the system. Vulnerability information is mainly derived from public vulnerability databases, including Common Vulnerabilities and Exposures (CVE), which cover important information, such as vulnerability numbers, vulnerability types, severity levels, attack complexity and exploitability, as shown in Table 2.

**Table 2:** Vulnerability information.

Name	Description	Example
vul_id	Vulnerability Id	“CVE-2023-27522”, “CNNVD-202201-1572”.
severity	Vulnerability severity level	Critical, High, Medium, Low
availability	The impact on the availability of the target system.	None (N), Partial (P), Complete (C)
confidentiality	The impact on the confidentiality of data processed by the system.	None (N), Partial (P), Complete (C)
integrity	The impact on the integrity of the exploited system.	None (N), Partial (P), Complete (C)
access	Difficulty of exploiting the discovered vulnerability.	High (H), Medium (M), Low (L)
exploitability	The exploitability scores of the vulnerability are based on baseMetricV3.	3.9
impact	The impact scores of the vulnerability are based on baseMetricV3.	3.6
base	The base scores of the vulnerability are based on baseMetricV3.It represent characteristics of the vulnerability itself, has three sub-groupings: Exploitability Metrics, Impact Metrics and Scope	7.5

The attack procedure information provides guidance on how to exploit the vulnerability, including recommended attack tools, operation procedures, and required conditions, as shown in Table 3.

**Table 3:** Attack step information.

Name	Description	Example
vul_id	Vulnerability Id associated with the attack step.	“CVE-2023-27522”, “CNNVD-202201-1572”.
step_no	Attack step number	1
step_info	Detailed information about the attack step.	Run the nmap tool to scan target ports.

Users can query, add, and modify vulnerability information and attack step information through this module. In addition, when new vulnerabilities are released, users or administrators can update the vulnerability library and attack steps through this module to keep the system’s vulnerability data synchronized and up-to-date. This design ensures that the system can adapt to the latest security threats and attack techniques in a timely manner.

### Vulnerability information parsing module

The vulnerability information analysis module processes input from vulnerability scans, analyzing and parsing the data. It identifies exploitable vulnerabilities in the system, then, based on network configuration, user permissions, and inference rules, determines all potential attack paths and their state transitions, as shown in Table 4. By using logical reasoning and path analysis, the module identifies possible attack chains, providing decision support for attack strategy development. Its output includes all attack transition states, paths, and node state information, supporting attack scheme generation.

**Table 4:** Attack state transition information.

Name	Description	Example
source	Source host	192.168.1.1
destination	Destination host.	192.168.1.2
vul_id	Vulnerability Id	“CVE-2023-27522”, “CNNVD-202201-1572”.
soft	Software with the vulnerability.	http_server_11.1.1.9.0
protocol	Network protocols exploited in the attack.	tcp
port	Port numbers exploited in the attack.	80

### Attack scheme generation module

The attack strategy generation module is responsible for generating customized attack strategies based on user-defined targets. To support diverse evaluation scenarios, the module provides extensive customizability beyond selecting the attack target. Users may control: (1) Path generation threshold, which limits the maximum number of returned attack paths; (2) Mandatory or forbidden node constraints (e.g., paths must include a certain host); (3) Vulnerability filtering criteria, such as minimum severity score, vulnerability type, or software category; (4) Scoring model selection, where users may choose between CVSS Base Score, Exploitability Score, Impact Score, or a weighted combination; (5) User-defined scoring functions, allowing replacement of the default CVSS-based prioritization with custom formulas.

After the path search is complete, the system provides attack step suggestions based on the vulnerability information of each host node in the generated path and finally generates a complete attack strategy. This module provides flexible control over path generation and condition constraints to ensure that the generated strategy meets the actual evaluation needs and provides highly customizable attack strategies for evaluators.

### Authentication module

The authentication module is a core security component of the system, responsible for implementing strict identity verification, fine-grained access control, and operation auditing to prevent unauthorized access and potential abuse. This module offers multi-factor identity authentication and role-based and attribute-based permission management to ensure that users can only access the functions necessary for their roles. Since the focus of this system is on generating attack schemes, only administrators and ordinary users have been set up. Administrators are responsible for system maintenance, configuration, and user management. Ordinary users can use the system functions normally and provide feedback on the execution effect of the generated solutions, assisting in the continuous correction of the model.

### User interaction visualization module

The user interaction visualization module is the user interface layer of the system, responsible for the input and output interaction of the system. This module presents the result of vulnerability analysis and the generated attack path in a graphical way, allowing users to intuitively view the attack diagram, path selection, and the attack status of each host node. Users can analyze each step of the attack scheme through this module and view the vulnerability information and attack details of each host node on the attack path. The design of the visualization interface allows users to quickly identify critical nodes and prioritize attack paths, providing intuitive support to optimize and decision making the attack scheme.

### Database

The database is the system data management module, responsible for storing the inference rules of the system, the vulnerability information, the analysis results, the attack schemes generated, and other parameters of the system configuration. The database uses structured storage to facilitate efficient retrieval and access of data. All data are stored in a standardized format in the database, including derivation rules, vulnerability information, attack paths, and attack schemes, which provide data support for the stability and consistency of the system. The database module also has backup and recovery functions to ensure the security and integrity of system data. Through the cooperation of the above modules, the system can provide a comprehensive and automatic attack path generation and verification platform for security testing and evaluation. The vulnerability information analysis module is responsible for analyzing and generating attack paths. The attack scheme generation module further optimizes and customizes the attack scheme. The overall framework of the system is not only suitable for security evaluation in complex network environments but also has good scalability and maintainability. It provides an efficient attack path generation and verification solution for security experts.

## Key technologies and implementation methods

This section introduces the key technologies and methods used in the attack scheme generation system. First, a network-wide simulation is set up for the target network to be evaluated. The vulnerability scanning tool is then used to gather vulnerability data, which, along with network configuration information, is input into the system for processing. The core technologies for parsing vulnerability information and generating attack schemes are critical for ensuring the system can efficiently generate and optimize attack paths, even in complex network environments. This section will focus on these two key aspects.

### Vulnerability information parsing technology

Vulnerability information parsing technology is one of the foundations of an attack scheme generation system. Its main function is to extract vulnerability information from nodes, services, and systems in the network, and input it into the attack graph generation module structurally for subsequent path generation and optimization. Since the vulnerability information formats of different nodes and systems are different, the core of the parsing technology is to standardize and structure the multi-source vulnerability data to make it suitable for the generation and update of attack graphs.

Our vulnerability information parsing process refers to the implementation method of the MulVAL system^20^. Specifically, the process parses vulnerability information based on derivation rules, which are in Datalog format, where variables start with uppercase letters and constants start with lowercase letters. Datalog is a simplified form of the Prolog language designed for knowledge bases and used for logical reasoning^21^. Each Datalog rule consists of three parts, the fundamental meaning of the rules is as follows:

1 $$\begin{aligned} P(X) \leftarrow Q(X, Y), R(Y), \text {condition}(X, Y). \end{aligned}$$

- *P*(*X*) represents the rule header, which usually a relational atom (such as student(x)) that represents the goal of the rule.
- *Q*(*X*, *Y*) and *R*(*Y*) represent the sub-goals in the body of a rule, representing preconditions. The entailment symbol “:-” can be read as “if” and is used to represent a logical relationship, not an operator.
- $\text {condition}(X, Y)$ represents the rule body, which consists of one or more sub-goals (e.g., $L_{1}, L_{2}, \ldots , L_{n}$), sub-goals can be relational atoms or Boolean-valued arithmetic expressions such as $a > 10$. The relationship between the sub-goals is equivalent to the logical “AND”.

The fundamental meaning of the rules is as follows. Traverse all the possible values of the variables, and the rule head holds when the variables satisfy all the sub-goals in the rule body. If the ruling body is empty, this rule is a “fact”; if the ruling body is not empty, it is called a “rule”.

The system sends the user-provided vulnerabilities, network configuration, and reasoning rules to the XSB engine for logical reasoning. Based on the relationships among vulnerability attributes, attacker privileges, and attack propagation paths, the XSB engine automatically deduces potential attack paths. After reasoning, the system processes the engine’s output to extract vulnerable hosts, associated vulnerability data, and attack state transitions between hosts. This information is then presented to the user through a visualization interface. In the visualization, hosts are represented as nodes, and clicking on a node displays the host’s vulnerability details. The directed edges between nodes indicate the transition paths of the attack, and clicking on an edge reveals detailed information about the attack, including vulnerability specifics, software name, protocol type, and port number.

**Figure 2:**
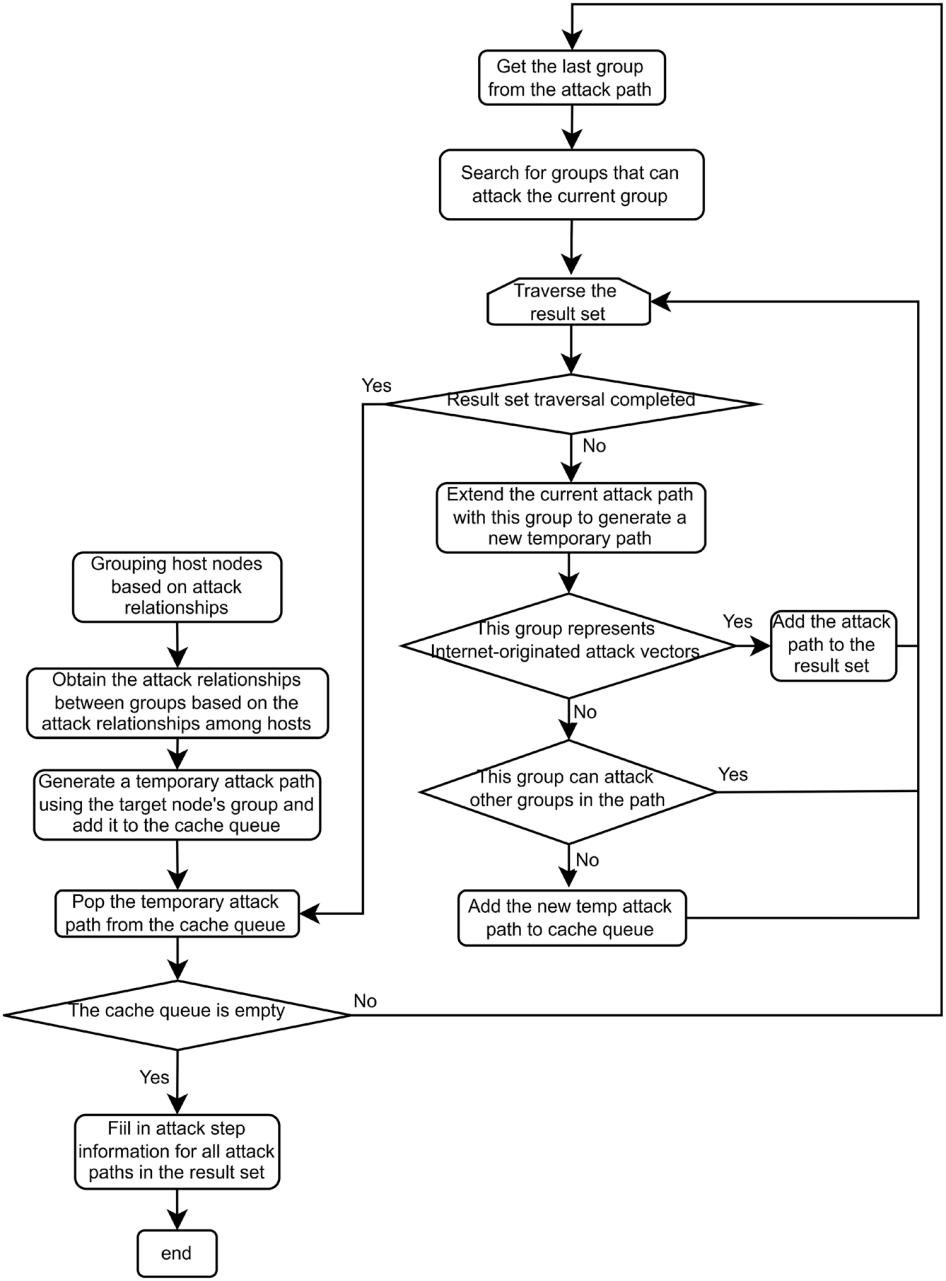
Attack scheme generation process.

### Method for generating attack schemes

The method of generating attack schemes constitutes the core of the attack graph generation system. Its objective is to generate feasible attack paths on the attack graph based on the network structure, node statuses, and vulnerability information of the system. Common approaches for generating attack schemes typically include heuristic search, path optimization, and intelligent algorithms. The system generates the optimal path via these methods, simulating the possible steps that an attacker might take, and facilitating network defense.The path ranking is initially based on the CVSS Base Score because it provides a standardized and widely accepted measure of vulnerability severity. However, users can customize the scoring function to incorporate other CVSS metrics (e.g., Impact Score, Exploitability Score) or even external metrics by adjusting the weights in $w_{i}$ the cumulative scoring formula:

2 $$\begin{aligned} S(P) = \sum _{i=1}^{n} w_i \cdot \textrm{Metric}(v_i) \end{aligned}$$

where $\textrm{Metric}(v_i)$ can be replaced or combined with other attributes such as CVSS Impact, Exploitability, or environmental scores. This flexibility allows evaluators to align path prioritization with organizational risk policies.

Upon completion of vulnerability information parsing, the system enables users to select specific host nodes and generate attack schemes for those nodes in real time. To avoid the path explosion phenomenon that may occur in a large-scale network and multi-vulnerability environment. The system adopts the way of grouping before searching, and sets a “path target threshold” to control the time and resource consumption of path generation. Once the number of generated paths reaches the threshold, the search will automatically cease. This threshold can be flexibly adjusted according to user requirements to avoid generating excessive redundant paths and ensure the accuracy of the assessment results.

Figure 2 illustrates the generation flow of the attack scheme. The whole process adopts the search strategy of “shortest path first”, and searches the reachable path from the target host to the source (i.e., the Internet) in reverse^29^. The steps are as follows:

Step 1: Host grouping. The hosts in the system are grouped according to the attack state transition information between hosts. The host nodes with the same attack and attacked node sets were divided into the same group, and the attack state transition information between groups was constructed according to the attack state transition information between host nodes.

Step 2: Initialize the attack path. First, create a temporary attack path from the target host node, fill the target host information into it, and store it in the cache queue as the starting point for the subsequent path search.

Step 3: The path search process. The attack paths to be processed is popped out from the cache queue in turn, obtains the last host node of the path, and searches according to the state information of the node to find all the attack state transition information that can reach the node.

Step 4: Group path generation. If there is a path whose source address is Internet in the state transition information, a packet attack path is generated.

Step 5: Grouping path expansion. If the packet corresponding to the source address in the state transition information does not appear in the current path, the packet will be appended to the end of the current path to generate a new temporary attack path and put back into the cache queue.

Step 6: Ranking vulnerability information. For the searched attack path, the vulnerabilities in each group are sorted in descending order according to the score of the corresponding vulnerability in the vulnerability knowledge base. The score of an individual vulnerability is obtained from the CVSS base score stored in the knowledge base. To evaluate the overall attractiveness of an entire attack path *P*, consisting of a sequence of exploited vulnerabilities $v_{1}, v_{2}, \ldots , v_{n}$, a cumulative scoring metric *s*(*P*) is used:

3 $$\begin{aligned} s(P)=\sum _{i=1}^{n} w_{i} \cdot \operatorname {CVSS}_{\text{ base } }\left( v_{i}\right) \end{aligned}$$

Where *P* represents a single attack path, which is an ordered sequence of steps; *n* represents the number of steps (vulnerability exploits) in the path P; $v_{i}$ represents the specific vulnerability exploited in the $i-th$ step of the path; $CVSS_{base}(\upsilon _{i})$ represents the CVSS Base Score of vulnerability $v_{i}$; $w_{i}$ represents a weighting factor applied to the score of the $i-th$ vulnerability.

Step 7: Initialize the cache queue. The attack path cache queue is created, and the queue is sorted by the path score from high to low. For each group attack path searched, the vulnerability with the highest score in each group was used to generate an attack path and join the cache queue.

Step 8: Attack path generation. The highest score path is extracted from the cache queue and the packet nodes of the path are traversed at the same time. For each node, if there are subsequent vulnerabilities in its group, it replaces the node with the next vulnerability to generate a new temporary path, calculates the total score and inserts it into the cache queue.

Step 9: Loop processing. The above steps are repeated continuously to check the constraints for each generated path. If the specified criteria are met, the path is added to the result set. The process ends until the cache queues are all processed or the number of attack paths found reaches the path target threshold.

Step 10: The scheme generation. The system traverses all generated attack paths in the result set. For each node in each path, the system will call the vulnerability information management module to obtain the detailed attack steps according to its vulnerability information, and gradually fill the final attack scheme.

The design process not only effectively controls the resource consumption of path generation, but also provides users with the flexibility to adjust the number of paths, so as to more efficiently meet the security evaluation requirements in different scale network environments.To substantiate the proposed method and ensure reproducibility, we provide the pseudo-code of the complete attack path generation algorithm. This algorithm incorporates host grouping, reverse search, path threshold constraints, and vulnerability-based ranking.

**Algorithm 1:**
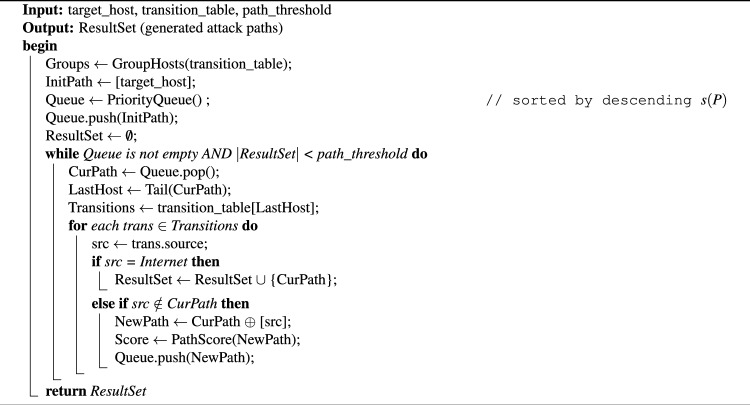
Attack Path Generation

The attack path search algorithm reduces the search space by grouping strategy, and adopts the “best path first” strategy to ensure that when the network scale is large and the number of attack paths far exceeds the set threshold, the best path is found first, so as to meet the actual needs of security evaluation more effectively^30^. Let *G* be the number of groups and P be the path threshold. The grouping step reduces the search space from $O\left( N^{2}\right)$ to $O\left( G^{2}\right)$ . The main loop runs at most *P* times, and each step explores up to *G* predecessors. Thus, the worst-case time complexity is $O(P \cdot G)$, which is linear in the number of groups and controlled by the threshold.

To improve processing efficiency, the system stored the attack state transition information for each node in the hash table with the sink node as the key in the preprocessing stage. In the search process, the system can directly search for the corresponding transition information of the attack state according to the current node, to achieve a query time complexity close to *O*(1), and significantly optimize the speed of path generation. After generating the attack scheme, the system shows the detailed attack path information to the user through the visual interface. You can view each attack step along the path in sequence, including the source node and destination node, the exploited vulnerability information, and the detailed attack operation of each step. This visual display provides users with an intuitive and systematic attack scheme, which is easy to analyze and make decisions.

### Self-evolution mechanism

The self-evolution mechanism refers to the system’s capability to dynamically and incrementally update the attack graph in response to changes in the operational environment, without requiring a full recomputation from scratch. This ensures the generated attack schemes remain current and relevant. The system continuously monitors the following data sources for changes:

- Vulnerability Databases: Periodic checks for new CVEs or updates to existing vulnerabilities.
- Network Configuration: Real-time monitoring of changes in network topology, host additions / removals, and service modifications.
- User-Defined Constraints: Updates to evaluation goals, path thresholds, or node filters.

When a change is detected, the system initiates the update process. The detection is implemented via a background daemon that compares the current state of the network and vulnerability database with the previously stored state. Changes are recorded and classified to determine the scope of required updates.

The update mechanism follows these steps:

Step 1: Change Identification: Identify the type and scope of the change (e.g., new vulnerability on a host, removed host, updated rule).

Step 2: Impact Analysis: Determine which parts of the existing attack graph are affected. This is done by querying the database for nodes and edges related to the changed elements.

Step 3: Partial Re-computation: Recompute only the affected subgraphs using the same reasoning rules and path generation algorithms, but applied incrementally.

Step 4: Graph Merge: Integrate the updated subgraph into the existing attack graph, ensuring consistency and removing obsolete paths.

Step 5: Validation: Verify the updated graph against the current network state and user constraints.

The incremental update process significantly reduces computational overhead compared to a full recomputation, especially in large-scale networks.

The following pseudo-code outlines the incremental update process:

**Algorithm 2:**
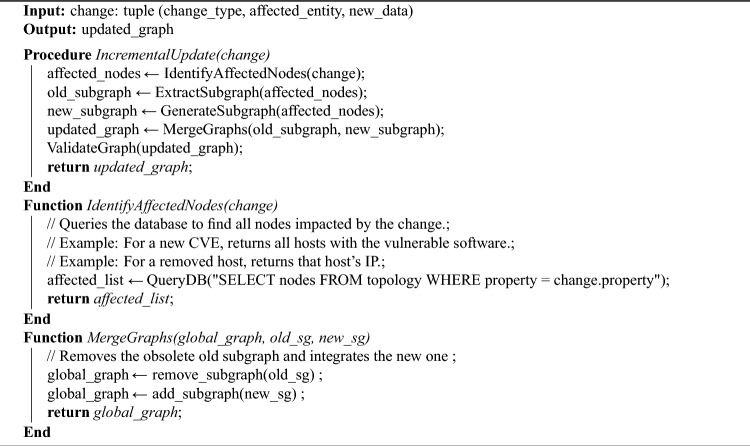
Incremental Update Process

## Experiment and evaluation

In this section, we describe the experimental cases, vulnerability scanning and analysis results, visual analysis of attack schemes, performance comparison with the MulVAL system, and comparative analysis of system processing efficiency in detail, which fully verifies the effectiveness and advantages of the proposed defense scheme generation system.

### Experimental scenario

**Figure 3:**
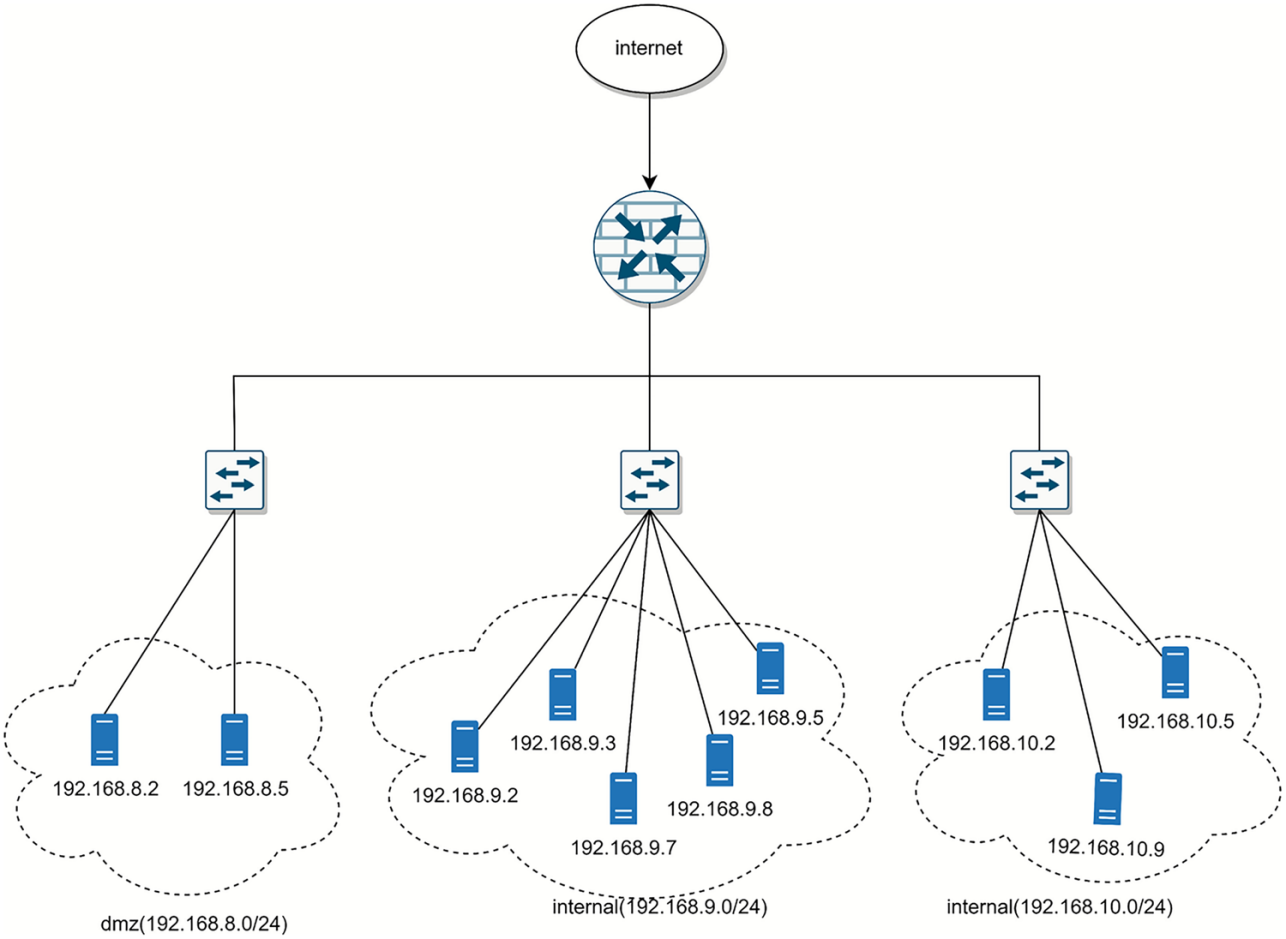
Example.

To evaluate the performance and effectiveness of the attack scheme generation system, we selected an enterprise network simulation scenario from a penetration testing and defense competition organized by Pengcheng Laboratory, which has been proven to be vulnerable to attacks. We then reconstructed an instance of this environment within a cyber range for experimental validation. The network topology is shown in Fig. 3, where the 192.168.8.0/24 network segment is divided into the DMZ area, which is used to provide external services. Two servers, with IP addresses 192.168.8.2 and 192.168.8.5, are deployed within this network segment to provide mail and web services, respectively. External access to these services is enabled through the configuration of Destination Network Address Translation (DNAT) policies on the router, which facilitates TCP-based connectivity to port 25 (SMTP) and port 80 (HTTP) for internet users. In addition, the 192.168.9.0/24 and 192.168.10.0/24 network segments serve as internal network areas where hosts within the same subnet can access all ports to each other. In the 192.168.9.0/24 network segment, the server with IP addresses 192.168.9.3 provides MySQL services, and the server with IP addresses 192.168.9.8 provides HTTP services. These services open ports 3306 and 80 to other hosts in the DMZ area and the inner network, allowing cross-subnet access.

**Figure 4:**
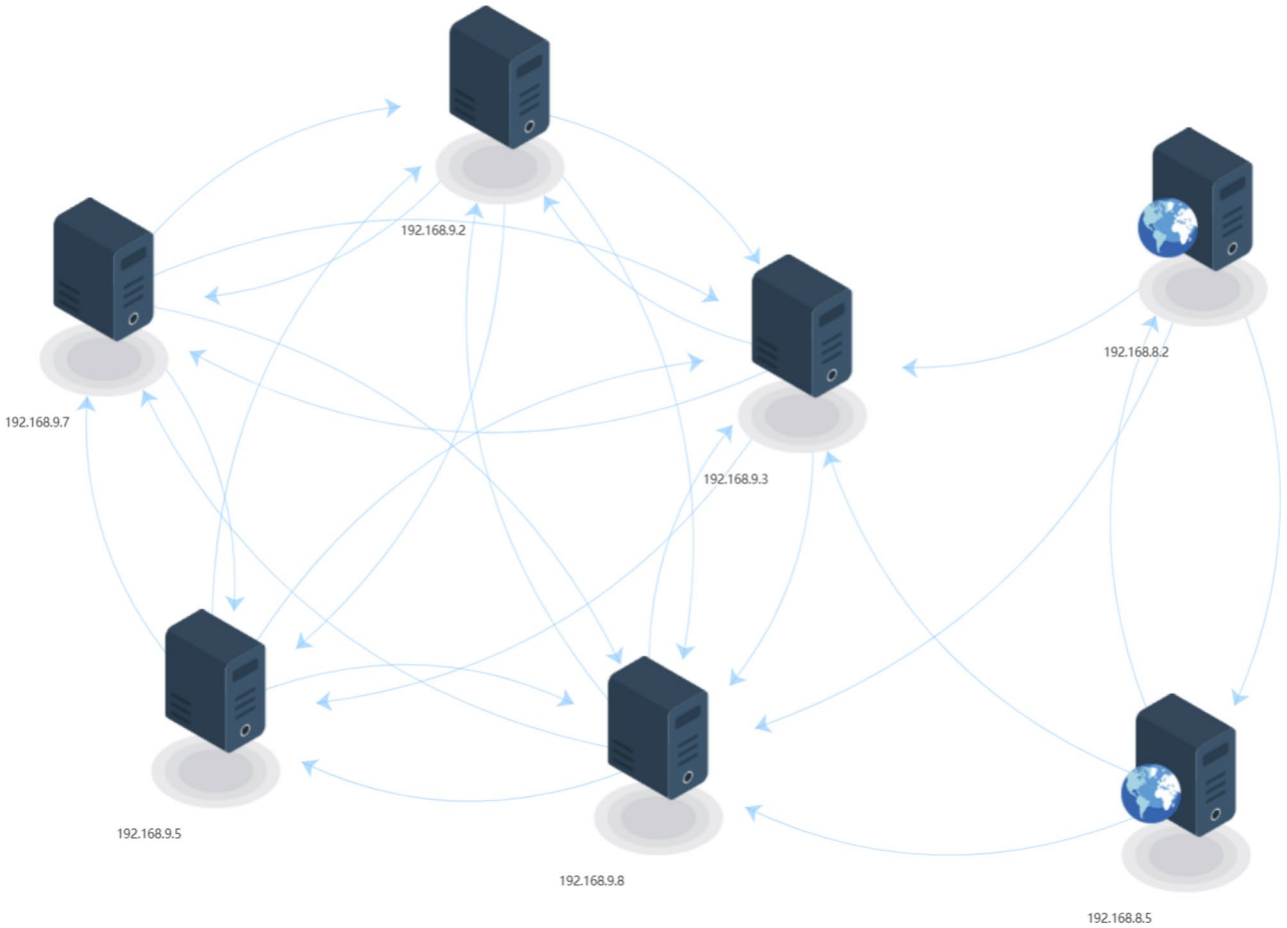
Attack reachability graph after parsing vulnerability information. Nodes represent hosts. Directed edges represent attack state transitions, indicating that an attacker can move from the source host to the destination host by exploiting a specific vulnerability.

In the simulated network environment, the vulnerability scanning tool is used to scan all hosts and collect vulnerability information from each host. After the scan is completed, the scan results are exported to a standard format and supplemented with the corresponding network configuration information according to the network topology. The vulnerability information found by each host is listed in Table 5. This vulnerability information is entered into the attack scheme generation system, and the graphical results of the attack reachable path are generated by the parsing function, as shown in Fig. 4.

**Table 5:** Vulnerability information.

Host	Vuln Id	Software	Protocol	Port
192.168.8.2	CVE-2013-2566	http_server_11.1.1.9.0	tcp	25
192.168.8.5	CVE-2023-25690	http_server_*	tcp	80
192.168.9.3	CVE-2016-6662	mysql_*	tcp	80
192.168.9.2	CVE-2016-2183	enterprise_linux_7.0	tcp	993
				995
192.168.9.5	CVE-2020-1350	windows_server_2008_r2	tcp	3389
				53
192.168.9.7	CVE-2013-2566	http_server_11.1.1.9.0	tcp	25
192.168.9.8	CVE-2023-25690	http_server_*	tcp	80
192.168.10.2	CVE-2016-2183	enterprise_linux_7.0	tcp	993
				995
192.168.10.5	CVE-2020-1350	windows_server_2008_r2	tcp	3389
				53
192.168.10.9	CVE-2015-2808	http_server_11.1.1.9.0	tcp	1433

As a comparison, the same vulnerability information is fed into the MulVAL system and the attack graph is generated. The results show that the reachable host information obtained by the two systems is consistent, which indicates that the accuracy of the attack path analysis of our system reaches the standard of the MulVAL system. The analysis results are visualized with the host as the node so that users can clearly see all the hosts that are reachable by the attack and the attack transition state between them. As can be seen in Fig. 4, there are a large number of potential attack paths between the host nodes in the same subnet and between the 192.168.8.2 and 192.168.8.5 servers to the 192.168.9.3 server. However, the 192.168.10.0/24 network segment does not open any external service ports, so no reachable attack path is detected in the system, and the host in this network segment does not appear in the parsing results. This parsing approach allows users to quickly identify at-risk host regions and understand potential attack paths for critical services in the subnet.

**Table 6:** Attack scheme generation statistics.

Destination	Attack Paths	Processing Time(ms)
192.168.8.2	2	0
192.168.8.5	2	0
192.168.9.2	432	2
192.168.9.3	188	1
192.168.9.5	432	1
192.168.9.7	368	2
192.168.9.8	188	1

### Attack scheme generation and detailed information visual analysis

The system allows the user to select the target host and generate the attack scheme for the target in real-time. Table 6 lists the statistics of attack schemes generated when different hosts are selected to help users understand the potential attack risks of each host. By clicking on each scheme entry, the user can view the detailed attack path information. Taking the target host 192.168.9.5 as an example, Table 7 lists the details of an attack scheme generated against this host. The attack scheme details include the name of the target, the number of hops in the attack path, and a list of the details of each step in the path. Each hop contains the node name of the source host and the target host, the exploited vulnerability number, the vulnerability description, the vulnerability impact score (e.g., integrity impact score, exploitability score), the access complexity, and the detailed attack steps suggestions. This kind of detailed information can help users fully understand each step of the attack process, which provides the basis for subsequent risk assessment and response strategy.

**Table 7:** An attack scheme example.

Target	192.168.9.2		
Hop Count	3		
1	Source	internet	
	Destination	192.168.8.5	
	Vul_Id	CVE-2023-25690	
	Software	http_server_*	
	Protocol	tcp	
	Port	80	
	Impact	5.9	
	Exp	3.9	
	Base	9.8	
	Access Complexity	Low	
	Description	Issue occurs when certain mod_proxy configurations on Apache HTTP server allow for HTTP request smuggling attacks.	
	Attack Steps	1	Identifying the CRLF Injection:Determine if the httpd version is less than 2.4.55, more information: https://owasp.org/www-community/attacks/HTTP_Response_Splitting.
		2	Internal HTTP Request Smuggling via Header Injection, more information: https://github.com/dhmosfunk/CVE-2023-25690-POC.
2	Source	192.168.8.5	
	Destination	192.168.9.3	
	Vul_Id	CVE-2016-6662	
	Software	mysql_*	
	Protocol	tcp	
	Port	3306	
	Impact	5.9	
	Exp	3.9	
	Base	9.8	
	Access Complexity	Low	
	Description	Some versions of MySQL allow local users to create arbitrary configurations and bypass certain protection mechanisms by setting general_log_file to a my.cnf configuration.	
	Attack Steps	1	Inject malicious configurations into MySQL configuration files with weak or improper permissions.
		2	Create new configuration files within the MySQL data directory on default MySQL installations without relying on improper configuration permissions.
		3	Gain access to logging functions on all of the default MySQL installations and thus be in position to add or modify config files.
3	Source	192.168.9.3	
	Destination	192.168.9.2	
	Vul_Id	CVE-2016-2183	
	Software	enterprise_linux_7.0	
	Protocol	tcp	
	Port	995	
	Impact	3.6	
	Exp	3.9	
	Base	7.5	
	Access Complexity	Low	
	Description	It easier for remote attackers to obtain cleartext data via a birthday attack against a long-duration encrypted session, as demonstrated by an HTTPS session using Triple DES in CBC mode, aka a “Sweet32” attack.	
	Attack Steps	1	Download attack tool script:
			git clone https://github.com/drwetter/testssl.sh.git
		2	Run the script with the target IP address as a parameter, for example :
			./testssl.sh -W 192.168.1.1.

### Performance comparison with the MulVAL system

With increasing network scale, the number of attack paths will increase exponentially, raising the requirements for path analysis and generation. By extending the 192.168.9.0/24 network segment and increasing the number of hosts, the processing time of the attack scheme generation system and the MulVAL system are compared, and the results are shown in Table 8. When the number of hosts increases to 100, the processing time of the MulVAL system increases dramatically to more than 1073 s, and the generated attack graph has many nodes, which is difficult to interpret, and the difficulty of finding specific attack paths is significantly improved. In the attack scheme generation system, the host node is used as the display unit, so even if the number of nodes increases, there is no node explosion phenomenon, and the at-risk hosts and their attack state transfer information can be clearly displayed. When the number of hosts is further increased, the running time of MulVAL is extended to more than ten hours, and the processing cannot be completed, while the attack scheme generation system can still stably provide clear parsing results.

**Table 8:** Processing time.

Hosts	Attack Scheme Generation System	MulVal
10	62ms	838ms
20	69ms	62s
50	158ms	264s
100	58s	1073s
200	71s	–
500	1339s	–

### The processing efficiency evaluation of the attack scheme generation system

**Figure 5:**
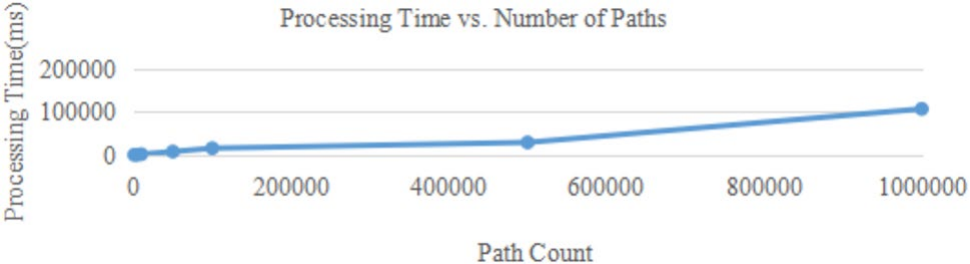
Processing time vs. number of paths.

In the analysis results shown in Fig. 4. 192.168.9.5 is selected as the target host, the attack scheme is generated by setting different threshold values of the number of paths, and the corresponding processing time is recorded. The results are shown in Fig. 5, where the processing time increases approximately linearly as the number of paths increases. When the number of attack paths is less than 10000, the processing time is less than 2 seconds, which meets the requirements of real-time. However, since the number of attack paths increases exponentially with the network size, the increase of network size will lead to uncontrollable processing time when there is no limit on the number of paths threshold. In the practical application of a large-scale network, a reasonable setting of the path threshold can effectively reduce processing time and improve the real-time performance and responseability of the system.

Although the current experiment scales up to 500 hosts, the system’s design–through grouping, thresholding, and incremental updates–ensures that computational complexity remains manageable even in larger networks. The linear time complexity observed in path generation (see Figure 5) suggests that the system can handle enterprise-scale networks (e.g., 10,000+ nodes) with appropriate threshold settings. However, to avoid exponential path explosion, we recommend setting a path threshold (e.g., 1,000 paths) based on the evaluator’s tolerance for processing time and resource constraints. Future work will include experiments on cloud-scale deployments with dynamic resource allocation.

In summary, our proposed attack scheme generation system exhibits good path generation, parsing, and visualization capabilities in the simulation environment, with higher parsing efficiency and clearer result presentation compared to the MulVAL system. When the network scale increases, the system can flexibly control the path generation scale by setting the threshold of the number of paths to ensure the practicability of the system in large-scale networks.

### Comparative analysis with existing approaches

To further validate the effectiveness and superiority of the proposed self-evolution attack scheme generation system, we conducted a comparative analysis with several existing approaches, including MulVAL^20^, a widely used logic-based network security analyzer, and other recent attack graph generation systems^6,19,31^. The comparison is based on the following criteria:

- Scalability: Ability to handle large-scale networks without exponential growth in processing time.
- Real-time performance: Time efficiency in generating attack paths.
- Visualization Support: Whether the system provides an intuitive graphical interface for path analysis.
- Customizability: Support for user-defined constraints and target-oriented path generation.
- Path Explosion Mitigation: Strategies to avoid redundant or irrelevant paths.

**Table 9:** Comparative analysis of attack graph generation systems.

Feature / System	Proposed System	MulVAL^20^	Boudermine^19^	Palma^25^	Nadeem^22^
Scalability	High	Low	Medium	High	Medium
Real-time Performance	Linear	Exponential	High	Medium	Medium
Visualization Support	Yes	No	No	Limited	No
Customizability	High	Low	Medium	Medium	Low
Path Explosion Mitigation	Threshold-based	None	Logic-based	Query-based	Alert-driven
Self-Evolution Mechanism	Yes	No	No	Partial	No

As shown in Table 9, the proposed system outperforms existing approaches in multiple aspects. While recent systems like Palma^25^ offer scalability and Nadeem^22^ enable alert-driven updates, our work integrates real-time interactivity, self-evolution, and human-in-the-loop evaluation in a single framework, addressing a gap in practical, usable AG tools for cybersecurity assessment. It supports real-time attack path generation with linear time complexity, provides an interactive visualization interface, allows user-defined constraints, and effectively mitigates path explosion through a threshold-based grouping strategy. Moreover, the self-evolution mechanism enables the system to adapt to dynamic network environments and updated vulnerability databases, which is not supported by any of the compared systems. These advantages make the proposed system particularly suitable for large-scale and complex network environments where efficiency, usability, and adaptability are critical for effective cybersecurity evaluation.

## Limitations and future work

While the proposed self-evolution attack scheme generation system demonstrates promising results in simulation environments, several limitations must be acknowledged, which also present opportunities for future research.

### Limitations

Dependency on Vulnerability Databases: The system relies heavily on external vulnerability databases (e.g., CVE) for up-to-date vulnerability information. Delays or inaccuracies in these databases could affect the timeliness and accuracy of the generated attack paths.

Scalability in Extremely Large Networks: Although the system incorporates grouping and thresholding mechanisms to mitigate path explosion, its performance may still degrade in ultra-large-scale networks (e.g., beyond 10,000 nodes) due to the exponential growth of potential attack paths.

Assumption of Static Network Topology: The current model assumes a relatively static network topology during the evaluation period. Dynamic environments with frequent changes in network configuration, host states, or access policies may reduce the relevance of generated attack paths.

Lack of Real-Time Adaptability: While the system supports real-time path generation based on user input, it does not yet fully integrate real-time network traffic analysis or live threat intelligence feeds, which could enhance the realism of attack simulations.

Generalization to Diverse Network Types: The system has been tested primarily on traditional IT networks. Its applicability to specialized environments such as IoT networks, industrial control systems, or cloud-native infrastructures requires further validation.

### Future research directions

To address these limitations and enhance the system’s practicality, the following research directions are proposed:

Integration with Real-Time Threat Feeds: Future versions could incorporate real-time threat intelligence and anomaly detection systems to dynamically update vulnerability profiles and attack rules, improving the system’s responsiveness to emerging threats.

Machine Learning for Path Prioritization: Leveraging machine learning techniques to predict the most likely attack paths based on historical data and attacker behavior models could further optimize path generation and reduce computational overhead.

Support for Dynamic Environments: Enhancing the system to handle dynamic network changes through continuous monitoring and adaptive graph updating would make it more suitable for real-world deployments.

Extended Validation in Diverse Domains: Conducting experiments in IoT, OT, and cloud environments would help to generalize the system and identify domain-specific challenges and solutions.

Human-in-the-Loop Evaluation: Incorporating feedback from cybersecurity experts during attack path evaluation could improve the system’s interpretability and practical utility in complex scenarios.

By addressing these challenges, the system can evolve into a more robust and widely applicable tool for proactive cybersecurity assessment.

## Conclusion

This paper proposes a self-evolution attack scheme generation system for testing and evaluation. The system can analyze the vulnerable host nodes in the network and the attack state transition information among them in real time after inputting the vulnerability information and network configuration information, and display it to the user through a graphical interface. The system supports users in selecting different host targets to automatically generate targeted attack schemes. To solve the problems of long processing time, insufficient real-time performance, and difficult interpretation of the results in traditional attack graph generation systems after increasing the network scale^31,32^. To prevent potential abuse or information leakage caused by automatically generated attack paths, we have designed an authentication module for user access control. Furthermore, we have masked sensitive host details in the output. The system is especially suitable for network security testing and evaluation, which provides support to testing personnel, helps generate system security testing schemes, evaluates potential risks, and proposes targeted security reinforcement strategies to fully enhance overall system security. In addition, the system can also be applied to security training scenarios to provide security personnel or learners with specific target attack scheme guidance, help them simulate real attacks step by step, and improve their practical ability.

## Data Availability

The datasets used and/or analysed during the current study available from the corresponding author on reasonable request.
